# In-hospital extracorporeal cardiopulmonary resuscitation: preliminary results in a second-level hospital

**DOI:** 10.5935/2965-2774.20230161-en

**Published:** 2023

**Authors:** Raimundo García-del Moral Martín, Manuel Muñoz Garach, Maria Eugenia Poyatos-Aguilera, Teresa Gil-Jiménez, Juan Caballero Borrego, Manuel Colmenero

**Affiliations:** 1 Intensive Care Unit, Hospital Universitario Clínico San Cecilio - Granada, Spain; 2 Department of Cardiology, Hospital Universitario Clínico San Cecilio - Granada, Spain

## INTRODUCTION

Cardiac arrest (CA) is a major health problem associated with serious personal and social consequences. In Spain, 50,000 CA cases are estimated to occur per year, half of which are expected to occur in health care facilities.^(1)^ The shortand long-term prognoses of these patients are associated with the early initiation of basic and advanced life support (ALS). The use of extracorporeal cardiopulmonary resuscitation (eCPR) is an alternative in some circumstances,^(2)^ and its results are also time dependent. The current indication for eCPR is refractory CA, defined by three unsuccessful defibrillation attempts or lasting more than 10 minutes.^(3)^ It could be cost-effective method for witness in-hospital CA, and immediate initiation of ALS and extracorporeal support are reported to have survival rates between 20 and 30%,^(4)^ which are closely related to low-flow time (time from CA to start of extracorporeal membrane oxygenation [ECMO] support). It has been proposed, that it is a highly complex technique, should be implemented in a center with high volume of cases and experience in the use of ECMO.^(5)^ However, centers with experience in the implementation of primary coronary intervention programs and the application of other mechanical support devices have characteristics that make the use of eCPR attractive, especially considering their proportion of personnel trained in cannulation of large vessels, witnessed CA, high-quality cardiopulmonary resuscitation (CPR) attempts, young patients with few comorbidities and short low-flow times. We present the preliminary results of an eCPR program in the catheterization laboratory in witnessed in-hospital CA.

## METHODS

The eCPR program starts on 3^rd^ March 2021 and was approved by the Hospital Service Management, complying with the requirements established by the Extracorporeal Life Support Organization (ELSO). Two programs were implemented in 2021, 3 in 2022 and 3 in the first six months of 2023.

The activation criteria were as follows: age less than 60 years, witnessed CA and known etiology. Activation was performed after 3 failed defibrillation attempts or 10 minutes of advanced life support. After considering the patient a candidate for eCPR, a mechanical cardio-compressor (LUCAS 3®) was used, and the patient was transferred to the Cardiac Catheterization Laboratory. The patient was cannulated and connected to the previously primed ECMO machine (Novalung ECMO System, Fresenius®). Vascular access was established percutaneously under ultrasound and fluoroscopic guidance. We used cannulas (Medtronic Biomedicus Nextgen) measuring 21F-55cm in length for venous drainage and 17F-18cm in length for arterial return in a femo-femoral configuration. The procedure was performed in coordination with the cardiologist and the referring ECMO intensivist. In all patients, a distal perfusion cannula (6F) was placed in the superficial femoral artery. Blood flow was established at 3lpm. After coronary angiography or thrombectomy, the patient was transferred to the intensive care unit for post-resuscitation care. If mechanical support is needed after the initial phase (first 48 hours), the patient is transferred to the reference center.

## RESULTS

Eight procedures were performed. In half of them, the eCRP team was alerted while the patients was in the Cardiac Catheterization Laboratory. Patient characteristics and clinical outcomes are described in table 1. The mean age of the patients was 55 years, and 62% were male. In 75% of cases, the diagnosis was acute myocardial infarction, and the initial rhythm was shockable. The mean time from low-flow time was 34 minutes (minimum 10, maximum 75). The mean ECMO support time in survivors was 47 hours. Three patients had flow problems during support related to loss of pulsatility. Three patients (37.5%) needed support for a period of more than 48 hours and were transferred. Favorable neurological outcomes, defined as cerebral performance category classes 1 - 2, occurred in 50% of patients (n = 4). The in-hospital survival rate was 37.5% (n = 3), and one of the recovered patient died suddenly (due to primary ventricular fibrillation) 72 hours after withdrawal of mechanical ventilation and circulatory support. The most common complication was bleeding, and 6 of the 8 patients needed transfusion of blood products. Two patients had severe hemorrhage due to vascular cannulation.

**Table 1:** Patient characteristics and clinical results

Characteristics	Patient 1	Patient 2	Patient 3	Patient 4	Patient 5	Patient 6	Patient 7	Patient 8
Demographics								
Sex	Man	Man	Man	Woman	Man	Man	Woman	Woman
Age (years)	58	54	59	51	58	55	57	51
CA and ALS								
Initial rhythm	VF	VF	VF	PEA	VF	VF	VF	PEA
Etiology	STEACS	STEACS	STEACS	PE	STEACS	STEACS	STEACS	PE
Location	ICU	Catheterization laboratory	Catheterization laboratory	Emergency department	Catheterization laboratory	Emergency department	Catheterization laboratory	ICU
Low flow time	75	18	27	45	35	34	30	10
Analytical data								
pH	6.9	7.18	7.24	7	7.15	7.22	< 6.8	7.02
Lactic acid (peak, mmol/L)	18.6	11.2	9	20	11.8	8	13.2	14
Length of time								
Time on ECMO (hours)	36	5 days	25	6	28 days	82	12	36
ICU stay (days)	1	13	10	1	48	13	1	8
Hospital stay (days)	1	13	26	1	48	17	1	18
Transfer	No	Yes	No	No	Yes	Yes	No	No
Survival								
ICU	No	Yes	Yes	No	No	Yes	No	Yes
Hospital	No	No	Yes	No	No	Yes	No	Yes
CPC	5	1	1	-	3	1	-	1
Cause of death	Brain death	Sudden death	Alive	Death on ECMO	WLS	Alive	Death on ECMO	Alive

CA - cardiac arrest; ALS - advanced life support; VF - ventricular fibrillation; PEA - pulseless electrical activity; STEACS - ST-segment Elevation Acute Coronary Syndrome; PE - pulmonary embo-lism; ICU - intensive care unit; ECMO - extracorporeal membrane oxygenation; CPC - cerebral performance category; WLS - withdrawal life support.

## DISCUSSION

**Figure 1:**
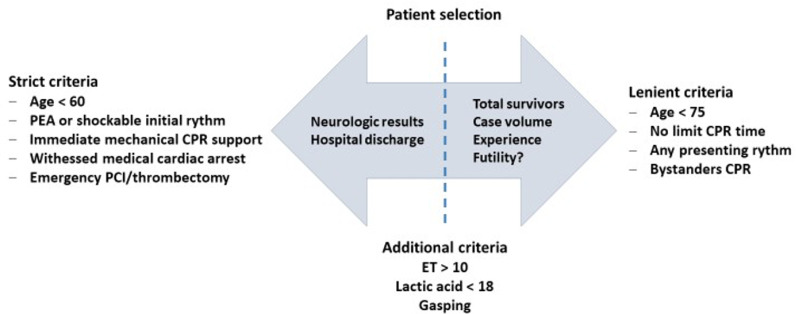
Impact of patient selection criteria on eCPR results and survival.

According to the ELSO, eCPR is defined as the application of veno-arterial ECMO to provide circulatory support when conventional CPR fails to restore sustained spontaneous circulation. It should be considered a rescue therapy for selected patients experiencing CA whose ALS was ineffective and to facilitate diagnostic and therapeutic interventions. In our case, six of the patients had acute coronary syndrome and underwent coronary angiography and coronary intervention, and in two patients, the cause of arrest was massive pulmonary embolism, and percutaneous thrombectomy was performed. There are currently no universally accepted inclusion and activation criteria. To start the program, we decided to optimize all factors associated with better outcomes in published studies (Figure 1).^(6)^

PEA - pulseless electrical activity; CPR - cardiopulmonary resuscitation; PCI - percutaneous coronary intervention; ET - end-tidal carbon dioxide.

Currently, there are three published series comparing the results of in-hospital eCPR with conventional CPR.^(7-9)^ The use of eCPR has been associated with increased survival rates, ranging from 23.5 to 31.3% (HR for eCPR 0.5 - 0.6; 95%CI 0.33 - 0.9). In Spain, one case series has been recently published. For one year, they performed 7 eCPRs, and the demographic characteristics, etiology, times and results were similar to those of our series.^(10)^ The difference between our hospital’s program and theirs is the absence of a Cardiac Surgery Service, and after both eCPR and the initial phase of post-resuscitation care, the patient is transferred to the reference center if there is no recovery of ventricular function and further assistance is required.^(11)^

The duration of basic and advanced life support prior to ECMO has been identified as a risk factor for an unfavorable outcome, with a cutoff point of 33 minutes for low-flow time.^(11)^ Given that cannulation can delay the procedure by 15 to 45 minutes, early activation of the eCPR team is essential. For conditions that require specific treatment interventions (acute myocardial infarction, pulmonary embolism), it is unlikely that CA will be resolved with ALS without these other interventions after the first 5 minutes,^(12)^ so the eCPR team should be alerted as early as possible after the third unsuccessful defibrillation attempt and the low-flow period should be shortened as possible.

## CONCLUSION

The extracorporeal cardiopulmonary resuscitation can be implemented in centers with experience in treating a selected group of patients with mechanical support, large vessel cannulation, and emergency primary coronary intervention/thrombectomy. The selection criteria must be strict at the start of the program.
